# Evolutionary history of an Irano-Turanian cushion-forming legume (*Onobrychis cornuta*)

**DOI:** 10.1186/s12870-024-04895-y

**Published:** 2024-03-20

**Authors:** Zahra Tayebi, Mahtab Moghaddam, Mohammad Mahmoodi, Shahrokh Kazempour-Osaloo

**Affiliations:** 1 Department of Plant Biology, Faculty of Biological Sciences, Tarbiat Modares University, Tehran, Iran 14115-154; 2 Botany Research Division, Research Institute of Forests and Rangelands, Agricultural Research, Education and Extension Organization (AREEO), P.O. Box 13185-116, Tehran, Iran

**Keywords:** *Onobrychis cornuta*, *O*. *Elymaitica*, Haplotype diversity, Irano-Turanian, Legume

## Abstract

The Irano-Turanian region is one of the largest floristic regions in the world and harbors a high percentage of endemics, including cushion-like and dwarf-shrubby taxa. *Onobrychis cornuta* is an important cushion-forming element of the subalpine/alpine flora of the Irano-Turanian floristic region. To specify the genetic diversity among the populations of this species (including individuals of *O*. *elymaitica*), we employed nrDNA ITS and two noncoding regions of plastid DNA (*rpl*32-*trn*L_(UAG)_ and *trn*T_(UGU)_-*trn*L_(UAA)_). The most striking feature of *O*. *cornuta* assemblages was the unexpectedly high nucleotide diversity in both the nDNA and cpDNA dataset. In the analyses of nuclear and plastid regions, 25 ribotypes and 42 haplotypes were found among 77 and 59 accessions, respectively, from Iran, Turkey, and Afghanistan. Network analysis of the datasets demonstrated geographic differentiation within the species. Phylogenetic analyses of all dataset retrieved *O*. *cornuta* as a non-monophyletic species due to the inclusion of *O*. *elymaitica*, comprising four distinct lineages. In addition, our analyses showed cytonuclear discordance between both nuclear and plastid topologies regarding the position of some *O*. *cornuta* individuals. The underlying causes of this inconsistency remain unclear. However, we speculate that chloroplast capture, incomplete lineage sorting, and introgression were the main reasons for this event. Furthermore, molecular dating analysis indicated that *O*. *cornuta* originated in the early Pliocene (around 4.8 Mya) and started to diversify throughout the Pliocene and in particular the Pleistocene. Moreover, *O*. *elymaitica* was reduced to a subspecific rank within the species.

**Supplementary Information:**

The online version contains supplementary material available at 10.1186/s12870-024-04895-y.

## Introduction

Cushion plants are generally compact, long-lived, low-growing, dome-shaped or mat-forming organisms. They can be found in very cold, very dry, or cold and dry habitats, and sometimes in warm and dry habitats worldwide [1–3]. Because of their often-domed shape, cushion species trap litter, increase soil quantity and nutrients, harbor microbial life for nutrient recycling, moderate temperature, store moisture, capture solar warmth, and act as wind shelters. Cushion plants are often considered nurse plants or facilitators of alpine habitats, providing a save rooting substrate for non-cushion species [3, 4].

Cushion-forming life is one of the most widespread evolutionary convergences, emerging at least 115 times in numerous clades of Angiosperms [2]. Fabaceae is one of the 62 families that contain the largest number of cushion-forming species belonging to several genera, such as *Astragalus* L., *Onobrychis* Mill. *Anarthrophyllum* Benth, and *Lupinus* L [1]..

*Onobrychis* has 205 accepted species [5], seven of which have a cushion life form [6]. Horned sainfoin is the most widespread cushion-forming species of the genus, distributed from West and Central Asia to Caucasus and N. Pakistan [5]. The nomenclatural history of the species was reviewed by Turland (1996) [7], who proposed that *H*. *cornutum* L., should be conserved against *H*. *spinosum* L., in which case the correct name and author citation would become *Onobrychis cornuta* (L.) Desv. [8], as already universally adopted [e.g., 6, 9, 10]. This taxon was established based on a single material collected by D. Gérard, from “the Oriente”- the unknown locality in the Middle East and Minor Asia- (Gérard 18 in Herb. Linn. No. 921.71; https://linnean-online.org/8094/#?s=0&cv=0). *Onobrychis cornuta* was classified as a member of *O*. sect. *Dendrobrychis* DC. [11], which was followed by subsequent treatments [6, 9, 12–14]. Based on recent molecular phylogenetic studies, its sectional position (as the type species of the section) was no longer tenable, and thus, along with its closest species, *O*. *elymaitica* Boiss. & Hausskn. transferred to *O*. sect. *Onobrychis* [15, 16]. *Onobrychis cornuta* has two accepted subspecies: subsp. *cornuta* and subsp. *leptacantha* Rech.f. They do differ in having strong spines vs. delicate spines, leaflet width > 1 mm vs. 0.5 mm, and fruit length of 6–12 mm vs. ± 5 mm, respectively [6, 17].

**Fig. 1:**
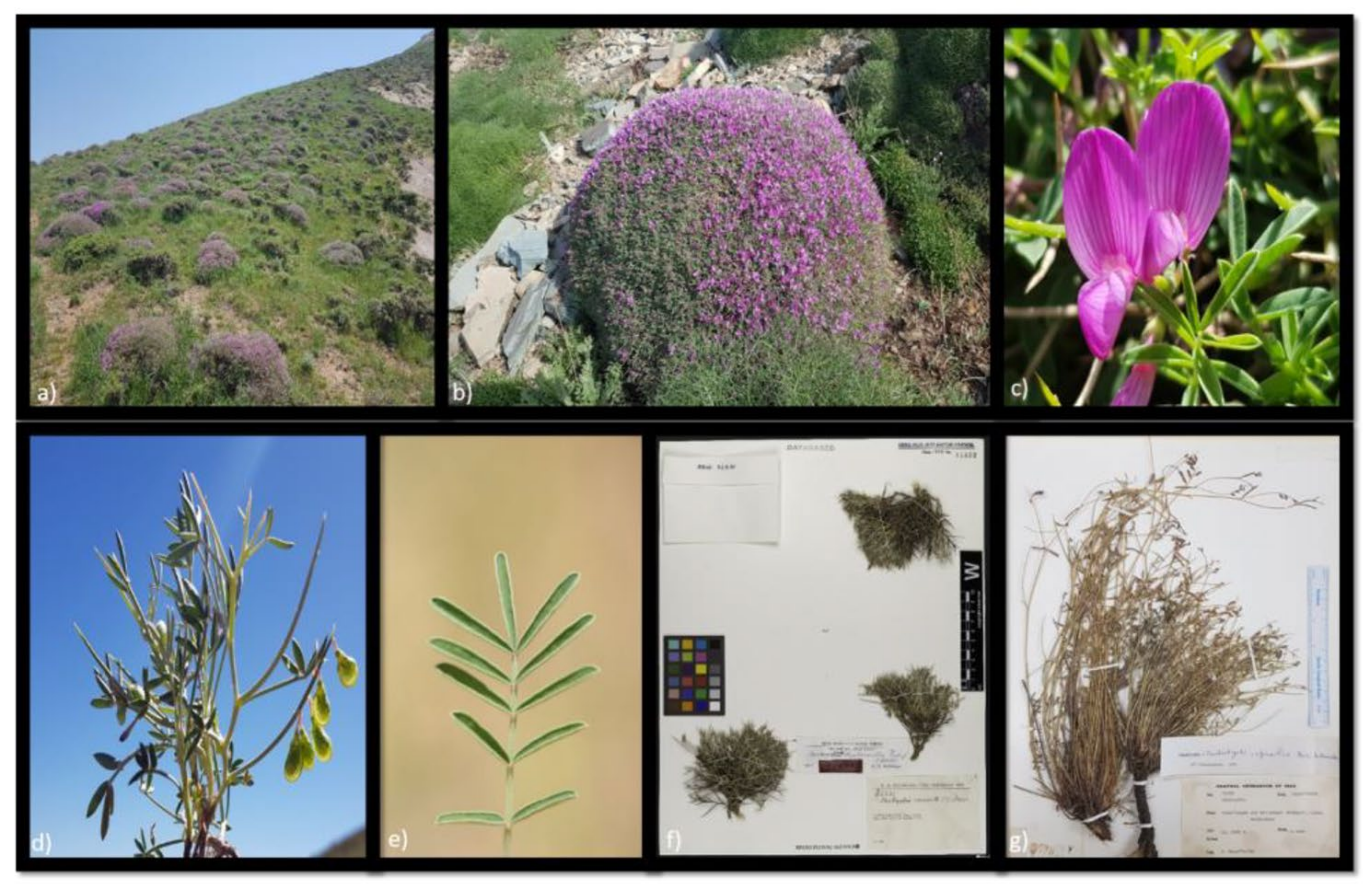
Representative of the *Onobrychis cornuta* species complex. (**a**) a community of the species, (**b**) an individual plant of the species in flowering stage, (**c**) a close up of flower, (**d**) an individual plant of the species in the fruiting stage, (**e**) a close up of leaves of *O*. *cornuta* with linear-lanceolate leaflets, (**f**) a view of the type material of *O*. *cornuta* subsp. *leptacantha* (https://www.jacq.org/detail.php?ID=478542) and (**g**) a view of herbarium specimen of *O*. *elymaitica* (photos by Z. Tayebi)

It is a densely twiggy, spiny shrub with a cushion-like habit up to 30 cm or more height and greater width with persistent spine-tipped peduncles and lax racemes of 3–6 flowers. This species is morphologically polymorphic in terms of the shape and size of the leaflets, density of the indumentum, and corolla size (Fig. 1a-f) [12, 6, pers. observ.].

This species, along with other congeneric species, has been studied from various perspectives, including gross morphology [e.g., 9, 6], karyology [18–21], fruit morphology [22], palynology [23, 24], genetic diversity [25], and molecular phylogeny [15, 16, 26]. Moreover, the species has been solely subject of the other research areas, such as community ecology [27], ecological niche modeling [28] and mountainous rangeland management [29, 30].

*Onobrychis cornuta* is an element of the Irano-Turanian (hereafter, IT) region, dominant in rocky mountain summits and dry rocky subalpine between 1200 and 3500 m. in elevation [12, 14]. This species is distributed across the mountainous region of Iran [6]. The IT region is one of the hotspots of biological diversity in the Old World and harbors cushion-like and dwarf-shrubby taxa [31, 32]. Several molecular phylogeny and phylogeographical studies have been conducted on cushion forming genera (e.g., *Dionysia* [33] particularly thorny genera from the IT region (e.g., *Acanthophyllum* [34, 35], *Acantholimon* [36–38] and *Astragalus* [39–41])).

Hitherto, no current comprehensive molecular phylogeny and phylogeographical studies of *O*. *cornuta* have been conducted. We used molecular markers (nrDNA ITS and two plastid intergenic regions: *rpl*32-*trn*L (UAG) and *trn*T(UGU)-*trn*L(UAA)) to address the following questions: (1) Does *O*. *cornuta* form a monophyletic group? (2) Are there distinct evolutionary lineages within this species? (3) Do phylogeographic patterns exist in the species?

## Results

### Phylogenetic analyses

The alignment of nrDNA ITS sequence for 80 accessions has 659 nucleotide sites, of which 70 were potentially parsimony informative (excluding outgroups).The aligned data matrices of *rpl*32-*trn*L_(UAG)_ and *trn*T_(UGU)_-*trn*L_(UAA)_ intergenic spacers for 68 and 57 accessions were 1014 and 1223 nucleotides long, respectively.

**Fig. 2:**
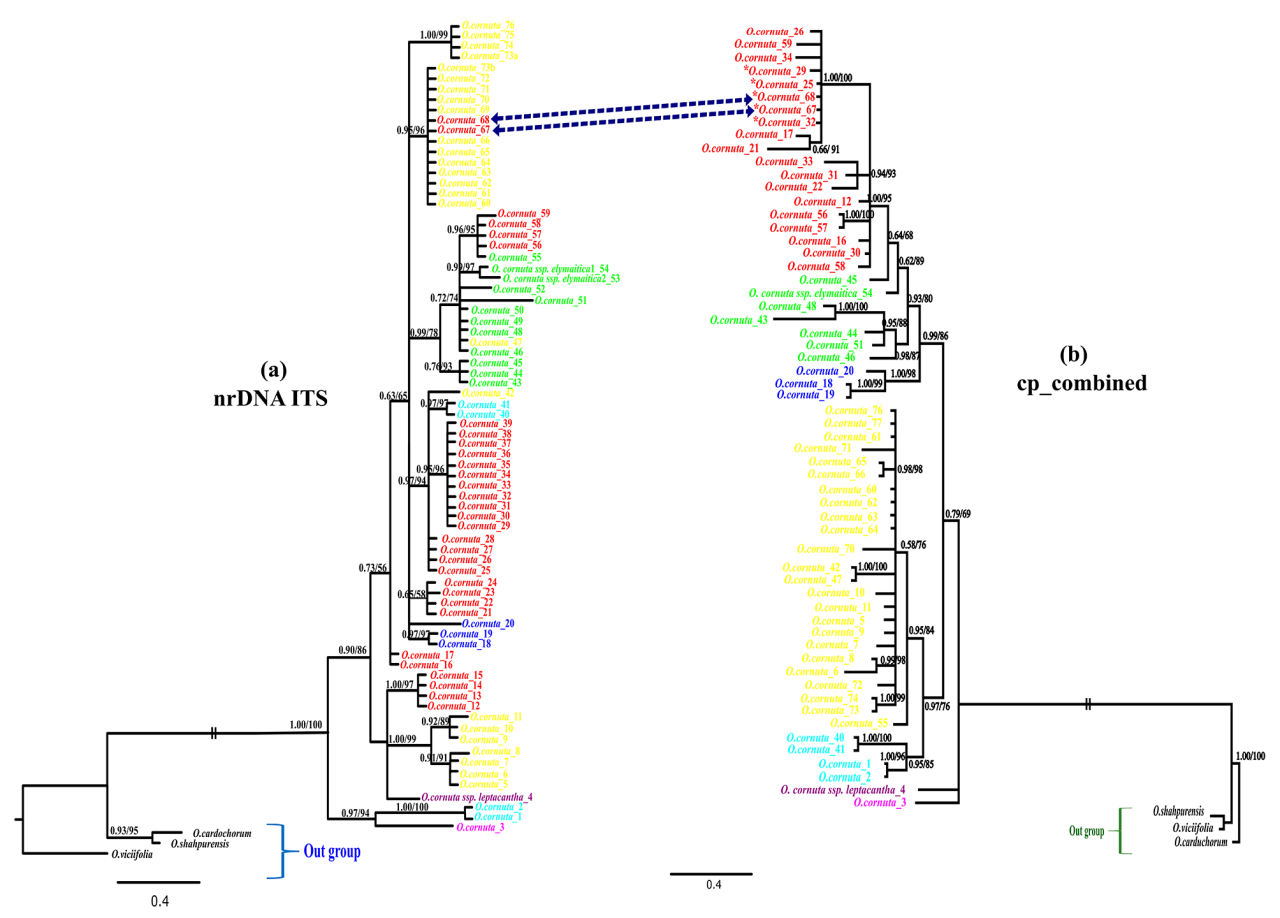
The 50% majority-rule consensus trees inferred from Bayesian analysis using (a) nrDNA ITS and (b) plastid combined dataset (*rpl*32-*trn*L_(UAG)_ and *trn*T_(UGU)_-*trn*L_(UAA)_) regions. Numbers above branches are the posterior probability (PP) of BI and bootstrap percentage (BP) of ML analysis, respectively. The accessions with dashed line are in conflict between trees a and b. The asterisk (*) represents accessions with an inversion of 8 bp in *rpl*32-*trn*L_(UAG)_ dataset

The number of parsimony informative sites was 29 and 39 for the first and second plastid regions, respectively, and the concatenated chloroplast dataset for 62 accessions had 2188 nucleotide sites, of which 118 were parsimony informative. Furthermore, the combined nuclear + plastid dataset for 49 accessions contained 2855 nucleotide sites, of which 147 were parsimony informative sites. Detailed descriptive statistics for the individual dataset (nrDNA ITS and plastid data) and the concatenated dataset are given in Table S2. Maximum likelihood (ML) and Bayesian inference (BI) analyses of the aligned data matrices (nrDNA ITS, cpDNA and nr + cp) yielded trees with the same topology. Thus, we used the Bayesian 50% majority-rule consensus tree topology and showed both posterior probabilities and bootstrap values on the branches. In the nrDNA ITS tree (Fig. 2a), two accessions from N Iran and one from Turkey formed the basal branches of *O. cornuta*, followed by a large assemblage of the remaining accessions. In this assemblage, *O. cornuta* subsp. *leptacantha* along with several accessions (form NE Iran and NW Iran) formed a sister group to several populations (including two accessions of *O*. *elymaitica*) from Central Alborz, NW Iran, and Zagros Mountains. In the plastid combined tree (Fig. 2b), two representatives of *O. cornuta*, including one individual from Turkey and *O. cornuta* subsp. *leptacantha*, are sisters to an assemblage of populations of the species (including *O*. *elymaitica*). Within this large clade, two accessions (no. 67 & 68), which belonged to the Central Alborz Mountain population in the nrDNA ITS tree, were well nested within a population from NW Iran in the plastid tree (Fig. 2).

**Fig. 3:**
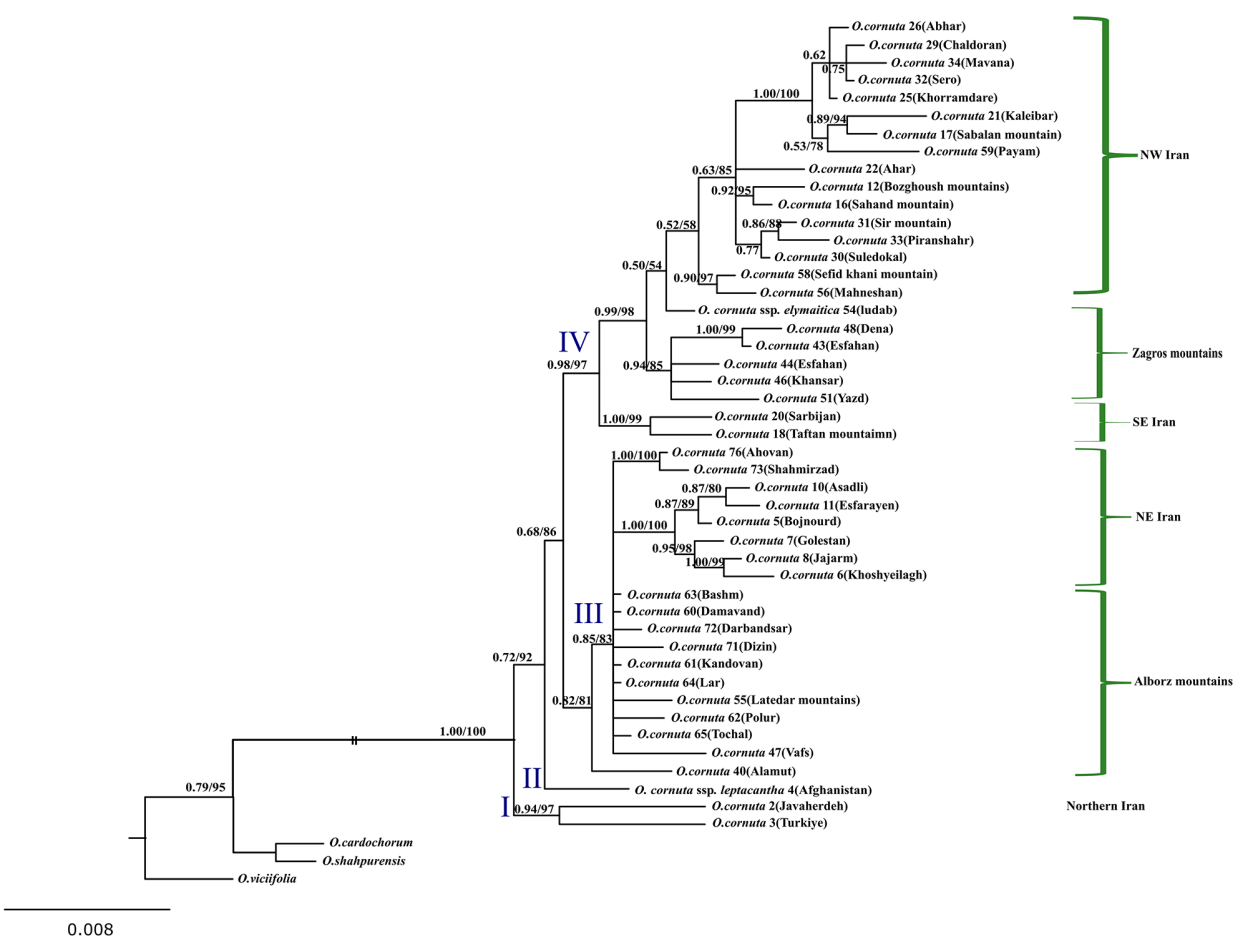
50% majority rule consensus tree from Bayesian analysis of the combined nrDNA ITS, *rpl*32-*trn*L_(UAG),_ and *trn*T_(UGU)_-*trn*L_(UAA)_ dataset. Numbers above branches are the posterior probability (PP) of BI and bootstrap percentage (BP) of ML analyses, respectively. Sample number and locality name for the accessions of *Onobrychis cornuta* were given beside its name

Analyses of the combined nr + cp. data demonstrated that *O. cornuta* consisted of four subclades. The first diverging subclade (“I”) comprised two individuals, one from Northern Iran (Javaherdeh) and another one from Turkey. *O. cornuta* subsp. *leptacantha* formed the second subclade (II), being sister to subclades “III” and “IV”. The subclade “III” comprised 19 accessions ranging from Central and Eastern Alborz to Northeastern Iran. The subclade “IV” is composed of 24 accessions (including *O*. *elymaitica*) ranging from Southeastern, through Zagros Mountains to Northwestern Iran (Fig. 3).

### Network analyses

**Fig. 4:**
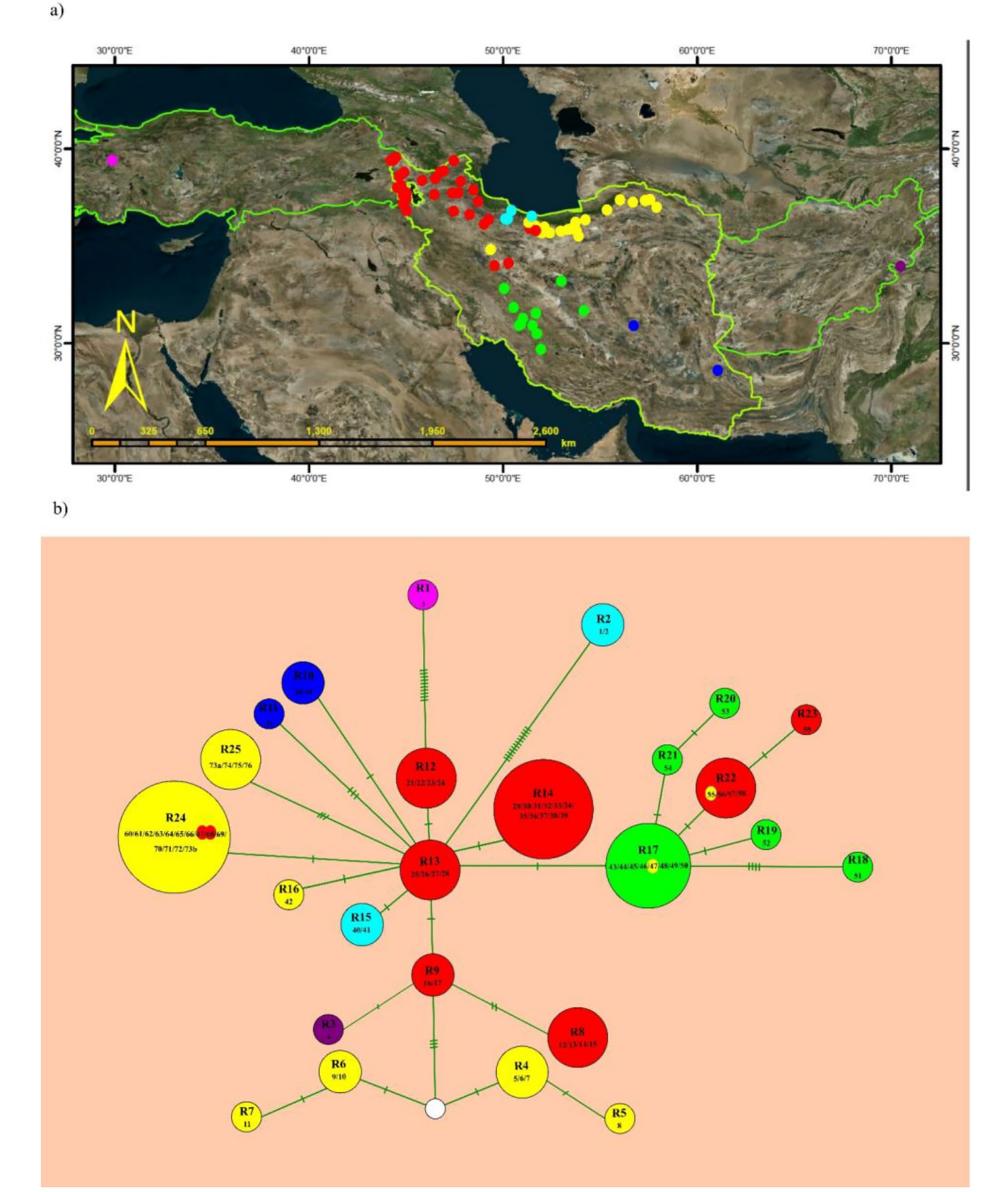
(**a**) Distribution of the nrDNA ITS-ribotype of *O*. *cornuta*, (**b**) nrDNA ITS ribotype network based on statistical parsimony. Circle size is proportional to the number of accessions in the ribotype. The map layout is prepared in the Arc GIS software environment (https://www.arcgis.com)

**Fig. 5:**
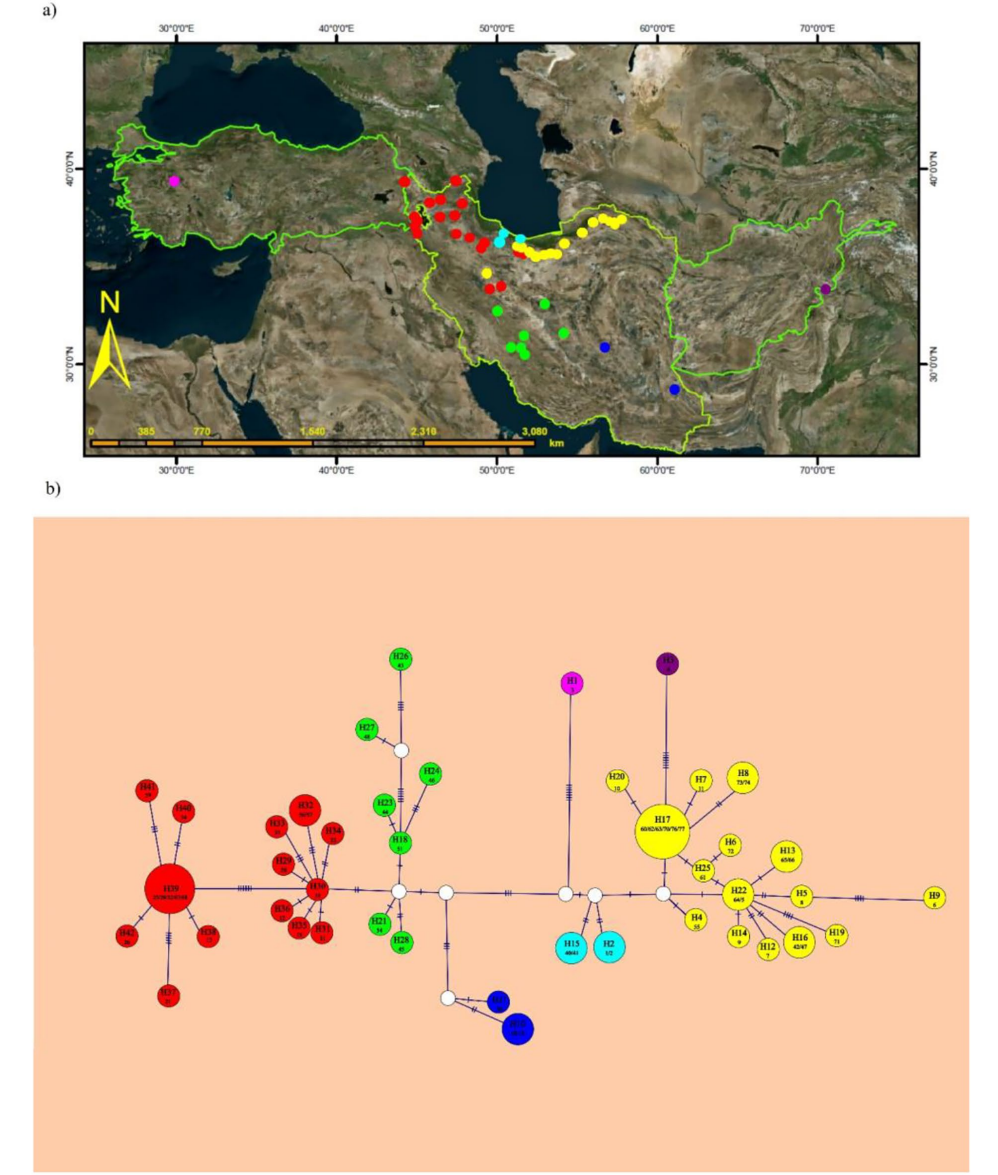
(**a**) Distribution of the cpDNA haplotype of *O*. *cornuta*, (**b**) cpDNA haplotype network based on statistical parsimony. Circle size is proportional to the number of accessions in the haplotype. The map layout is prepared in the Arc GIS software environment (https://www.arcgis.com)

Of the 77 accessions of *O. cornuta*, 25 ITS haplotypes (ribotypes) were detected with a frequency ranging from one to 14 individuals. Nucleotide sequences of the concatenated cpDNA were obtained for 59 accessions, revealing a total of 42 distinct haplotypes. These haplotypes represent remarkable genetic diversity within the species, particularly with varying frequencies ranging from a single to multiple individuals. Figures 4 and 5 show an unrooted haplotype network using statistical parsimony for both datasets. The basic statistics of haplotypes of each DNA regions are presented in Table 1. The nuclear and plastid data set displayed both negative values for Tajima’s D and Fu and Li’s F*, implying that the populations experienced no bottleneck.

### Divergence time estimation

**Fig. 6:**
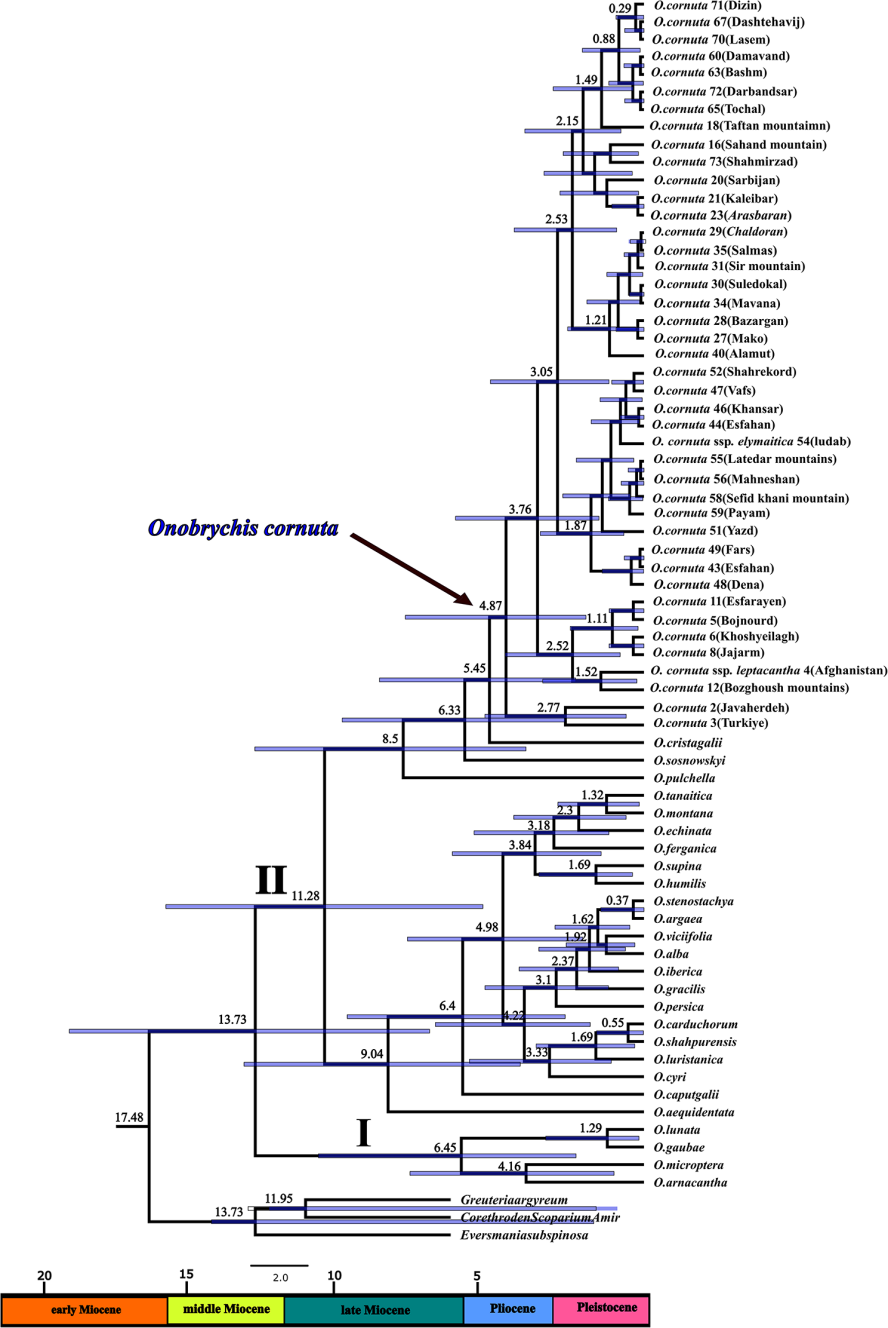
Chronogram inferred from BEAST anaylsis of nrDNA ITS. Each node represents the mean divergence time estimate and blue bars represent the 95% highest posterior density intervals around mean nodal ages. Sample number and locality name for the accessions of *Onobrychis cornuta* were given beside its name

Results from the BEAST analysis of the nrDNA ITS dataset presented in Fig. 6 revealed that the effective sample size (ESS) of all parameters greater than 500.

Our analysis indicated that the stem-node (*Greuteria*/*Eversmannia*/*Corethrodendron* + *Onobrychis*) was estimated to be the Early Miocene (∼ 17.4 Mya). In addition, the most recent common ancestor (MRCA) of *Onobrychis*, which is divided into two major clades (I and II), dated to the Middle Miocene (∼ 13.7 Mya). Clade I comprises representative species of *O*. subgen. *Sisyrosema* dated to the Late Miocene (∼ 6.4 Mya), and clade II is composed of representative species of *O*. subgen. *Onobrychis* with special reference to *O. cornuta* populations diverged by the Middle Miocene (∼ 11.2 Mya). Our dating analysis indicated that *O. cornuta* originated (∼ 4.8 Mya) during the early Pliocene and diversified throughout the Pliocene and Pleistocene.

## Discussion

### Taxonomic status and phylogenetic relationship within ***O***. ***cornuta***

*Onobrychis cornuta* exhibits significant morphological character polymorphism among other *Onobrychis* species across its distribution range [12, 6, pers. observ.].

Based on our concatenated dataset (nr + cp), different individuals of *O. cornuta* formed four lineages. (Fig. 3). With the exception of lineage ”I”, other three ones demonstrate geographical differentiation. Lineage I which comprised accessions from N Iran and Turkey, well diverged from the rest of *O. cornuta*, (at least materials from Turkey) and was morphologically distinct (having five - nine leaflet pairs and villose indumentum) from others. *O. cornuta* subsp. *leptacantha*, as lineage II, is distinguished by some features including leaflets width of 0.5 mm, delicate spiny peduncles and tiny fruits (5 mm) and restricted to Afghanistan and Pakistan [6, 17]. The lineage “III” includes individuals restricted to Central & Eastern Alborz and NE Iran, which is distinct in possessing oblong-elliptic leaflets with 4–6 mm long as well as shorter standard (10–12 mm. Finally, lineage “IV” mainly comprising specimens with linear-lanceolate leaflets of 10–25 mm longer standard (13–19 mm) and confined to Zagros Mountain, NW to SE Iran.

*O. elymaitica* is well nested within this lineage and distinct from *O. cornuta* in having some autapomorphic features including longer internodes, calyx teeth longer than calyx tube and multiflowered racemes (6–10 flowers) and limited distribution range in SW Iran (Fig. 1g) [6]. Moreover, the specific rank of this taxon is no longer tenable and herein reduces to the subspecies rank. Given that, we provisionally propose a diagnostic key to the infra-specific of *O. cornuta* (see Taxonomic treatment). However, to determine the exact taxonomic status of these taxa, additional studies are needed in the future.

### Haplotype network

Analysis of nuclear data revealed the existence of 25 distinct ribotypes among 77 individuals, indicating a substantial level of ribotype diversity within *O*. *cornuta*. The ribotype network structure showed that two ribotypes from Turkey and northern Iran (R1 and R2) were genetically distant from the remaining ones, with 11 and 12 mutations, respectively, which also formed the early diverged lineages in the nrDNA ITS tree (Fig. 2a). Surprisingly, R1 (corresponding to H1), in contrast to R2 (H2), also had the highest number of mutations in both the plastid tree and haplotype network (seven mutations), indicating that this might be an ancient haplotype/lineage within *O*. *cornuta* (Figs. 4b and 5b). Ribotype R13 was located at the center of the haplotype network (as an internal ribotype), whereas the other ribotypes were radially arranged, with distances of 1–3 mutations (Fig. 4b). Because the distances between ribotype R13 and most of the other derivative haplotypes were not large, these haplotypes probably diversified rather recently.

The internal ribotype (R13) is associated with individuals displaying linear-lanceolate leaflets of up to 25 mm in length, which in some haplotypes have changed to ovate leaflets. Interestingly, there have been instances where ovate leaflets have reversed the linear characteristics observed in the ancestral state. For instance, R13, with linear-lanceolate leaflets, has evolved into R17 which possesses ovate leaflets and subsequently undergone a reversal in R22 and R23.

Despite these differentiations, some derived haplotypes (R14, R12, R9, R8, and R3) retained ancestral linear-lanceolate leaflets within the taxon. The haplotype network analysis of nrDNA effectively demonstrated geographic differentiation within the species. The segregation of haplotypes in the network was almost similar to their position in the nrDNA ITS phylogenetic tree (Fig. 2a).

Haplotype network analysis of chloroplast data, comprising 59 accessions and 42 distinct haplotypes, revealed that the majority of haplotypes had a single individual. H39 and H17 exhibited internal haplotypes, represented by five and six individuals, respectively. Among all the plastid haplotypes, the H30 haplotype, restricted to NW Iran, experienced more genetic diversity than other haplotypes. The chloroplast haplotypes, similar to the nuclear haplotypes, demonstrate almost the same geographic differentiation. The haplotype networks and phylogenetic trees do not provide evidence for the recognition of *O. elymaitica* (R21, H21) as a distinct species from *O. cornuta*, although it is somewhat morphologically different.

Our findings revealed that the *O*. *cornuta* complex has greater genetic diversity than its consectional (*O*. sect. *Onobrychis* sensu Amirahmadi et al. 2016 [15]) species such as *O*. *viciifolia* [42, 43] and *O*. *transsilvanica*/*O*. *montana* [44]. In the present study, the most remarkable feature of *O*. *cornuta* complex has unexpectedly high haplotype and nucleotide diversities in both nrDNA and cpDNA dataset (Table 1). However, these indices have been found to be low in some other species (e.g. *Oxytropis chakaensis* [45], *Iberis simplex* [46]). The occurrence of high haplotype and nucleotide diversity within *O*. *cornuta* accessions could be explained by its life form (woody spiny shrub), breeding system (outcrossing) as well as the widespread geographical distribution [47, 48].

**Table 1:** Summary of genetic diversity indices and results of neutrality tests (Tajima’s D and Fu and Li’s Fs) for nuclear and chloroplast data. H, number of haplotypes; Hd, haplotype diversity; π, nucleotide diversity

	H	Hd	π	D	Fs
ITS	25	0.9421	0.01361	-2.02881	-5.872
cpDNA	42	0.9795	0.01	-1.87955	-15.403

### Divergence time

The utilization of BEAST analysis has provided valuable insights into the origin and subsequent diversification of *Onobrychis* in the context of the climatic conditions during the Middle Miocene through the Pleistocene, which is consistent with the time estimation of previous studies [16, 49] (Fig. 6). As mentioned above, *O*. *cornuta* is one of the most important components of Irano-Turanian region. The IT region, as one of the largest floristic regions in the world [31, 32], underwent cooling and aridification around the Middle Miocene, which were the results of the tectonic events (e.g. uplift of the mountains ranges (Alborz, Caucasus, Kopet Dagh, Pamir, Taurus, Tian Shan and Zagros) and plateau regions (Anatolian, Iranian and Qinghai-Tibetan)) [50–52, 32].

*O*. *cornuta*, as a cushion-forming element of subalpine/alpine region, originated around 4.8 Mya in early Pliocene and started to diversify throughout Pliocene and in particular Pleistocene (Fig. 6). The diversification of the *O*. *cornuta* assemblage can be explained by Pleistocene glaciation and geologic events. Pleistocene climatic oscillations (glacial-interglacial episodes) actively promoted diversification. In glacial periods, the level of habitat connectivity was increased, thereby affecting gene exchange between isolated populations and prompting allopatric speciation, while in interglacial periods, the isolation between alpine habitats was enhanced due to the development of climax vegetation in temperate zones [53]. On the other hand, alpine plant radiation, which is considered recent and rapid, occurred in all the main mountain ranges of the world in the Pliocene and Pleistocene [54–56]. The genetic differentiation that leads to the emergence of new species/populations conforming to specific environmental conditions is facilitated during Pleistocene fluctuations [57, 58]. Notable examples of rapid radiation in this era have been well-ducumented in some cushion-forming taxa (e.g. *Acantholimon* [38]; *Acanthophyllum* [34, 35] and thorny cushion *Astragalus* [59]).

### Cytonuclear discordance

Different natural factors (such as hybridization, introgression, chloroplast capture, and incomplete lineage sorting) have been proposed to explain the discordance between nuclear and organellar phylogenies [60]. One of the most likely reasons for the inconsistency between paternal and chloroplast DNA-based phylogenetic trees is the chloroplast capture. Chloroplast capture, which is the introgression of chloroplast from one species (population) into another, occurs when cytoplasmic substitution has an advantage in seed production [61–64].

Phylogenetic trees and haplotype networks based on nrDNA ITS and cpDNA data are topologically incongruent regarding the position of some individuals: (I) Two individuals from Central Alborz (67–68 with ribotype R24) do share the same plastid haplotype (H39) with a population from NW Iran. H39 is an internal and relatively old haplotype, and thus, incomplete lineage sorting may be a more plausible explanation for two distant populations sharing the same cp. haplotype [65, 66]. Furthermore, haplotype H39 and its derived haplotypes (H37-38 and H40-H42) are distinguished by an inversion of 8 bp in the *rpl*32-*trn*L_(UAG)_ region (Fig. 2b). These two individuals are morphologically (having ovate leaflets) more similar to the paternal population (ribotype R24) than to the maternal population. It is supposed that the pods of an ancestral maternal population due to high viability were dispersed via biological agents, such as birds and herbivorous mammals, toward Central Alborz and established therein in the past [67]. (II) Another case of discordance between nrDNA ITS and cpDNA was detected for ribotypes R4-R7 restricted to NE Iran. The ribotypes in both nrDNA ITS phylogeny and network (Figs. 2a and 4a) formed a distinct lineage, while in the cpDNA tree and network was nested along with Central Alborz population H17 and H22. Members of this group are geographically close to each other, and a possible hypothesis for explaining the cytonuclear discordance can be either introgression or incomplete lineage sorting (ILS). However, it was difficult to distinguish between these events in our case. Generally, recent divergence, large population size, and shallow bifurcation patterns are factors that indicate the occurrence of ILS as the reason for the inconsistency between gene trees [66]. In contrast, the populations of NE Iran and Central Alborz have almost sympatric distribution; in this case, incompatibility cannot be attributed to the ILS. NE haplotypes (H5, H9, H11, H12, H14, H20) may have captured their chloroplasts through introgression from the Central and Eastern Alborz haplotypes (H17 and H22, Fig. 5) (see also the next section).

III) The last is a local hybridization event that we found in an individual (no. 73) restricted to the eastern central Alborz, Shahmirzad, Semnan province). In the nrDNA ITS, four unambiguous polymorphic sites (A/C, C/T, T/C, G/T) were detected, which are the product of hybridization between ribotype R24 as a paternal plant and ribotype R25 (corresponding to plastid H8) as maternal one, both growing in the same region. This hybrid may have recently evolved because nrDNA ITS has not yet undergone concerted evolution.

### Taxonomic treatment

*Onobrychis cornuta* (L.) Desv., J. Bot. Agric. 3:81 (1814) sensu Tayebi.

Type: Habitat in Oriente, Gérard 18 in Herb. Linn. No. 921.71 (https://linnean-online.org/8094/#?s=0&cv=0).

= *Hedysarum cornutum* L., Sp. Pl., ed. 2.: 1060 (1763).

= *Hedysarum spinosum* L., Syst. Nat., ed. 10: 1171. nom. rej. prop.

= *Dendrobrychis cornuta* (L.) Galushko, Novosti Sist. Vyssh. Rast. 13: 251 (1976).

*O*. *cornuta* subsp. *leptacantha*.

Type: Afghanistan, prov. Jaji, inter Dre Kalla et Qasim Khel, 11 July 1965, Rechinger 32,331 (W; designated by Negaresh et al. 2022).

*O. cornuta* subsp. *elymaitica* (Boiss. & Hausskn.) Tayebi & Kaz. Osaloo, com. nov.

Type: Iran: Kuh-i Nur ad Tang Nalli, 2100–2400 m, Hausskneckt s.n. (W).

Syn: *O. elymaitica* Boiss. & Hausskn.

### Key to the subspecies of O. *cornuta*

1a. Plant with long internodes, leaflets linear-lanceolate, spiny peduncles delicate, calyx 6–7 mm, teeth longer than tube, (SW Iran) *O*. *cornuta* subsp. *elymaitica*.

1b. Plant with short internodes, leaflets variable, spiny peduncles stout or delicate, calyx 3.5-5 mm, teeth shorter than tube 2.

2a., Leaflet linear-lanceolate, 5–8 × 0.5 mm, spiny peduncles delicate, calyx c. 4 mm long (Afghanistan and Pakistan) *O*. *cornuta* subsp. *leptacantha*.

2b. Leaflet elliptic-oblong or linear-lanceolate 7–25 × 1–3 mm, spiny peduncles stout, calyx 4.5–6 mm long (across the species range) *O*. *cornuta* subsp *cornuta*.

## Conclusions

This is the first study on the phylogeny and distribution patterns of *O. cornuta* nuclear ribotypes and plastid haplotypes across a large part of the IT floristic region. The present study revealed high genetic diversity among accessions of this species in both the nuclear and plastid regions. The species is phylogenetically composed of four lineages and is not monophyletic due to the inclusion of *O. elymaitica* as its morphologically closest relative. Our findings indicate that *O*. *cornuta* originated in the early Pliocene (4.8 Mya) and diversified across the Pliocene and Pleistocene. The species has undergone cytonuclear discordance in some distantly and closely related entities, which might be caused by ILS or chloroplast capture and subsequent introgression events. Finally, the data obtained from this study could be a framework for further research on the phylogeography/genetic structure of the species across its distribution range.

## Materials and methods

### Sampling and DNA sequencing

In the present study, 77 accessions of *O*. *cornuta* and *O. elymaitica* were selected for molecular studies, of which 35 were collected by us from different habitats in various localities almost throughout Iran between 2020 and 2022. The specimens were deposited in the Tarbiat Modares University Herbarium (TMUH). The leaves of 42 remaining samples were obtained from various herbaria: Ferdowsi University of Mashhad Herbarium (FUMH), Gazi University Herbarium (GAZI), Herbarium of Isfahan Agricultural and Natural Resources Research and Education Center (SFAHAN), Museum of Natural History Vienna (W), Herbarium of Research Institute of Forests and Rangelands (TARI), Tehran University Herbarium (TUH), Herbarium of University of Isfahan (HUI) and West Azerbaijan Natural Resources Research Center Herbarium (WANRCH) (Table S1). The materials were identified by Sh. Kazempour-Osaloo and Z. Tayebi.

Total genomic DNA was extracted from dried leaf materials using the Doyle and Doyle CTAB method [68] with slight modifications. The nrDNA ITS region (ITS1-5.8 S-ITS2) was amplified by using AB101 and AB102 as the forward and reverse primers, respectively [69]. Also, two cpDNA intergenic spacers, including *rpl*32-*trn*L_(UAG)_ (using primers rpl32-F and trnL^(UAG)^ [70]) and *trn*T_(UGU)_-*trn*L_(UAA)_ (using primer pair trna and trnb of Taberlet et al. [71] as well as using the newly designed forward primer in this study: trnT-F (5′-ATCAATTGTGTGTGCATGCAT-3′) were used in this study.

PCR amplification was performed for all regions within a microtube containing 8 µl deionized water, 10 µl of 2 × Taq DNA polymerase master mix Red (Amplicon), 0.5 µl of each primer (10 pmol/µl), and 1 µl of template DNAFor nrDNA ITS region, the PCR program was 4 min at 94˚C for predenaturation followed by 33 cycles of 1 min at 94˚C for denaturation, 1 min at 55˚C for primer annealing and 1 min at 72˚C for primer extension, followed by a final primer extension of 7 min at 72˚C. PCR procedures for cpDNA regions were 4 min at 94˚C for predenaturation followed by 35 cycles of 1 min at 94˚C for denaturation, 1 min and 20 s at 55˚C for primer annealing and 1–2 min at 72˚C for primer extension, followed by a final primer extension of 7 min at 72˚C. PCR products were separated by electrophoresis in 1% agarose gels in 1 × TBE buffer (pH = 8) stained with ethidium bromide. PCR products using the appropriate primers were sent for Sanger sequencing to Pishgam Inc.

### Phylogenetic analyses

The best nucleotide substitution model for each locus was estimated using jModelTest [72] implemented in the Phylemon 2.0 web-server [73] based on the Akaike information criterion (AIC). Sequences were aligned using the online version of MAFFT [74] and adjusted manually. We conducted Baysian analyses of the dataset using MrBayes ver.3.2 [75] as implemented in CIPRES Science Gateway [76] at https://www.phylo.org. The maximum likelihood analyses were performed using the online phylogenetic software W-IQ-TREE [77] available at http://iqtree.cibiv.univie.ac.at. *Onobrychis carduchorum* C.C.Towns., *Onobrychis shahpurensis* Rech.f. and *Onobrychis viciifolia* Scop. were chosen as outgroups according to Hadadi et al. [16]

### Genetic diversity and haplotype analyses

The determination of haplotype/ribotype diversity was carried out based onthe statistical parsimony using the TCS networking method implemented in the POPART (Population Analysis with Reticulate Trees) software program [78]. For each dataset (nrDNA ITS and cpDNA), haplotype diversity (Hd) and nucleotide diversity (π) were estimated. To detect departures from the standard neutral model of evolution in the ITS and cpDNA combined (*rpl*32-*trn*L_(UAG)_ + *trn*T_(UGU)_-*trn*L_(UAA)_) dataset, we performed Tajima’s D [79] and Fu and Li’s Fs [80] tests using DnaSP v.6.12 [81]. To test for a correlation between geographic and genetic distances, we performed a Mantel test [82] using GenAlEx 6.5 software [83].

### Estimation of divergence time

To estimate the divergence times of the *Onobrychis* clade, we used the powerful phylogenetic tool, BEAST ver. 1.10.4 [84] on the CIPRES Science gateway. Because of the absence of reliable fossils of the genus and its relatives in the IR-loss clade, our analyses were performed based on secondary calibration using age estimates from previous studies [49, 85, 86]. The clock was calibrated using the estimate of mean age 15.69 ± 3 Mya for a node, encompassing the genus *Onobrychis* [49, 86]. In this study, an uncorrelated relaxed clock model was selected. Analyses were performed for 10 × 10^6^ generations with a burn-in of 10%. The Yule model was used as a tree prior. The convergence of parameters was checked visually and via effective sample sizes (to be at least 200) using Tracer 1.7.2 [87].

### Electronic supplementary material

Below is the link to the electronic supplementary material.

Supplementary Material 1

Supplementary Material 2

## Data Availability

Annotated sequences are publicly available from DDBJ (http://getentry.ddbj.nig.ac.jp/) under LC792040-LC792234 accession numbers (see Table S1).

## Abbreviations

IT: Irano-Turanian
