# Supplementary material for: Evolutionary history of an Irano-Turanian cushion-forming legume (Onobrychis cornuta)
Source: BMC Plant Biol. 2024 Mar 20;24:204. doi: 10.1186/s12870-024-04895-y (PMC10953250; doi:10.1186/s12870-024-04895-y)
Supplement: Supplementary file 1 — Supplementary Material 1 [file 12870_2024_4895_MOESM1_ESM.docx]

**Table S1** Taxa included in the nrDNA ITS, *rpl*32-*trn*L_(UAG)_ and *trn*T_(UGU)_-*trn*L_(UAA)_ analyses.

| GenBank accession no.  nrDNA ITS/*rpl*32-*trn*L_(UAG)_/ *trn*T_(UGU)_-*trn*L_(UAA)_ | Haplotype/  Ribotype | DNA source (location, voucher) | Species |
| --- | --- | --- | --- |
| LC792090/LC792156/LC792225 | H38/R9 | Iran: Ardebil, Meshkinshahr, Sabalan mountain, Ramezani 2020-1 (TMUH) | *Onobrychis cornuta* (L.) Desv. 17 |
| LC792107/LC792169/- | -/R8 | Iran: Ardebil, Mozaffarian & Nowroozi 34320 (TARI) | *O. cornuta* 15 |
| LC792077/LC792173/LC792211 | H35/R9 | Iran: East Azarbayejan, Bostanabad, Sahand mountain, Ramezani 2020-2 (TMUH) | *O. cornuta* 16 |
| LC792084/LC792150/LC792219 | H36/R8 | Iran: East Azarbayejan, Mianeh, Bozghoush mountain, Tayebi 2020-3 (TMUH) | *O. cornuta* 12 |
| LC792094/-/- | H36/R8 | Iran: East Azarbayejan, Mianeh, Bozghoush mountain, Ramezani 2020-33 (TMUH) | *O. cornuta* 13 |
| LC792076/LC792143/LC792210 | H34/R12 | Iran: East Azarbayejan, Ahar, Assadi 86685 (TARI) | *O. cornuta* 22 |
| LC792096/-/- | -/R12 | Iran: East Azarbayejan, Arasbaran, Kaleibar, Assadi & Vosughi 24903 (TARI) | *O. cornuta* 23 |
| LC792097/LC792159/- | -/R12 | Iran: East Azarbayejan, Arasbaran,Kakeibar, Assadi & Maassoumi 20183 (TARI) | *O. cornuta* 24 |
| LC137028^*^/LC137115^*^/LC792215 | H41/R23 | Iran: East Azarbayejan, Marand, Payam, Kazempour Osaloo et al. 2012-2 (TMUH) | *O. cornuta* 59 |
| LC792075/LC792142/LC792209 | H37/R12 | Iran: East Azarbayejan, Kaleibar, Bahadori 2020-4 (TMUH) | *O. cornuta* 21 |
| LC792100/LC792162/- | -/R14 | Iran: West Azarbayejan, Urmia, Khanghah-Sorkh, Bahadori 2020-07 (TMUH) | *O. cornuta* 39 |
| LC792088/LC792154/LC792223 | H40/R14 | Iran: West Azarbayejan, Urmia, Mavana, Bahadori 2021-8 (TMUH) | *O. cornuta* 34 |
| LC792086/LC792152/LC792221 | H31/R14 | Iran: West Azarbayejan, Urmia, Sir mountain, Kazempour Osaloo & Jarchi 2021-9 (TMUH) | *O. cornuta* 31 |
| L/C792087/LC792153/LC792222 | H33/R14 | Iran: West Azarbayejan, Piranshahr, Mashkan mountain, Ghasempoor & Larti (WANRCH) | *O. cornuta* 33 |
| LC792081/LC792147/LC792216 | H30/R14 | Iran: West Azarbayejan, Sule-Dokal, Bahadori 2021-6 (TMUH) | *O. cornuta* 30 |
| LC792104/LC792166/- | -/R13 | Iran: West Azarbayejan, mountains west of Bazargan, Assadi&Mozaffarian 30168 (TARI) | *O. cornuta* 28 |
| LC792091/-/- | -/R14 | Iran:West Azarbayejan,Salmas, Alizadeh&Khodakarimi 4434 (WANRCH) | *O. cornuta* 35 |
| LC792092/-/- | -/R14 | Iran: West Azarbayejan,Salmas, Alizadeh&Heidari7288 (WANRCH) | *O. cornuta* 36 |
| LC792102/LC792164/- | -/R13 | Iran:West Azarbayejan, Maku, Alizadeh&Ghasempour 4555 (WANRCH) | *O. cornuta* 27 |
| LC792093/-/LC792228 | -/R14 | Iran: West Azarbayejan, Khoy,Badalan Alizadeh 6846 (WANRCH) | *O. cornuta* 37 |
| LC792083/LC792149/LC792218 | H39/R14 | Iran: West Azarbayejan,Sero Kazempour Osaloo & Jarchi 2021-5 (TMUH) | *O.cornuta* 32 |
| LC792082/LC792148/LC792217 | H39/R14 | Iran:West Azarbayejan,Chaldoran, Alizadeh & Ghasempour 6025 (WANRCH) | *O.cornuta* 29 |
| LC792101/LC792163/- | -/R14 | Iran: West Azarbayejan, Khoy, Garehziyaadin Alizadeh 6901 (WANRCH) | *O. cornuta* 38 |
| LC792095/-/- | H32/R22 | Iran:Zanjan, Mahmoodi 98721 (TARI) | *O. cornuta* 57 |
| LC792089/LC792155/LC792224 | H32/R22 | Iran:Zanjan, Mahneshan,Tayebi 2020-10 (TMUH) | *O. cornuta* 56 |
| LC792085/LC792151/LC792220 | H42/R13 | Iran:Zanjan,Abhar, Jarchi 2021-11 (TMUH) | *O. cornuta* 26 |
| LC792078/LC792144/ LC792212 | H39/R13 | Iran:Zanjan,Khorramdareh, Jarchi 2021-12 (TMUH) | *O. cornuta* 25 |
| LC792057/-/- | H15/R15 | Iran:Ghazvin,Alamut, Akbar-Abad Foroughi 754 (TARI) | *O.cornuta* 40 |
| LC792056/LC792124/LC792191 | H15/R15 | Iran: Ghazvin,Alamut, Tayebi 2020-13 (TMUH) | *O. cornuta* 41 |
| LC792045/LC792118/LC792185 | H17/R24 | Iran:Tehran, Poloor, Tayebi 2020-14 (TMUH) | *O. cornuta* 62 |
| LC792046/LC792126/LC792193 | H22/R24 | Iran:Tehran,Lar, Tayebi 2020-15 (TMUH) | *O. cornuta* 64 |
| LC792049/-/- | H17/R24 | Iran:Tehran,Lasem,Tayebi 2020-16 (TMUH) | *O. cornuta* 70 |
| LC792047/LC792158/LC792227 | H13/R24 | Iran:Tehran,Tochal, Tayebi 2020-17 (TMUH) | *O. cornuta* 65 |
| LC792048/-/- | H13/R24 | Iran:Tehran,Tochal, Tayebi 2020-32 (TMUH) | *O. cornuta* 66 |
| LC792050/ LC792127/LC792194 | H17/R24 | Iran:Tehran, Damavand Assadi & Mozaffarin 33171 (TARI) | *O. cornuta* 60 |
| LC792054/ LC792122/LC792189 | H6/R24 | Iran:Tehran,Darbandsar, Tayebi 2020-18 (TMUH) | *O. cornuta* 72 |
| -/LC792117/LC792184 | H17/- | Iran:Tehran,Damavand, Tayebi 2020-19 (TMUH) | *O. cornuta* 77 |
| LC792051/LC792119/LC792186 | H19/R24 | Iran:Tehran,Dizin, Tayebi 2020-20 (TMUH) | *O. cornuta* 71 |
| LC792044/LC792116/LC792183 | H25/R24 | Iran:Karaj Kandovan, Riazi 2174 (TARI) | *O. cornuta* 61 |
| LC792079/LC792145/LC792213 | H39/R24 | Iran:Lavasan, Dashte-Havij, Kazempour Osaloo 2021-21 (TMUH) | *O. cornuta* 67 |
| LC792080/LC792146/LC792214 | H39/R24 | Iran: Lavasan, around Latian Dam, Kazempour Osaloo 2021-22 (TMUH) | *O. cornuta* 68 |
| LC792098/LC792157/LC792226 | H29/R22 | Iran:Markazi, Arak, Sefid-Khani Mountains, Tayebi 2021-23 (TMUH) | *O. cornuta* 58 |
| LC792055/LC792123/LC792190 | H4/R22 | Iran:Markazi, Arak, Latedar mountains, Mozaffarian 63824 (TARI) | *O. cornuta* 55 |
| LC792106/LC792168/- | H16/R17 | Iran:Markazi, Komijan Vafs, Tayebi 2021-24 (TMUH) | *O. cornuta* 47 |
| LC792058/LC792128/LC792195 | H16/R16 | Iran: Markazi,,Komijan, Vafs mountain, Tayebi 2021-34 (TMUH) | *O. cornuta* 42 |
| LC792068/LC792171/- | H10/R10 | Iran:Taftan, Mozaffarian 53066 (TARI) | *O. cornuta* 18 |
| LC792067/LC792136/LC792203 | H10/R10 | Iran:Taftan, Mokhtari 2021-25 (TMUH) | *O. cornuta* 19 |
| LC792108/ LC792114/LC792181 | H2/R2 | Iran:Ramsar, Javaher-Deh, Runemark & Maassoumi 20849 (TARI) | *O. cornuta* 2 |
| AB329699^*^/-/LC792230 | H2/R2 | Iran: Mazandaran Kheirud-Kenar, Kazempour Osaloo 2006-5 (TMUH) | *O. cornuta* 1 |
| LC792065/LC792134/LC792201 | H12/R4 | Iran:Gorgan, Golestan National Park, Foroughi 5538(TARI) | *O. cornuta* 7 |
| LC792105/LC792167/- | -/R8 | Iran:Gilan, Masuleh to Khalkhal, Assadi 86462 (TARI) | *O. cornuta* 14 |
| LC792069/LC792137/LC792204 | H24/R17 | Iran:Esfahan, Khansar, Mokhtari 2021-26 (TMUH) | *O. cornuta* 46 |
| LC792071/LC792139/LC792206 | H26/R17 | Iran:Esfahan, Nowroozi & Etemadi 1316 (SFAHAN) | *O. cornuta* 43 |
| LC792070/LC792138/LC792205 | H23/R17 | Iran:Esfahan, Nowroozi 4093 (SFAHAN) | *O. cornuta* 44 |
| LC792072/LC792161/- | H28/R17 | Iran:Esfahan, Aryavand 4621(HUI) | *O. cornutab* 45 |
| LC792073/LC792140/LC792207 | H27/R17 | Iran:Yasuj Dena Mountain, Bahadori 2021-27 (TMUH) | *O. cornuta* 48 |
| LC792099/LC792160/- | -/R19 | Iran:Shahrkord, Mozaffrian 54876 (TARI) | *O. cornuta* 52 |
| LC792103/LC792165/- | -/R17 | Iran:Fars, Abadeh, Foroughi 17344 (TARI) | *O. cornuta* 49 |
| LC792064/LC792133/LC792200 | H9/R4 | Iran:Shahrud, Khoshyeilagh, Wendelbo et al. 11163 (TARI) | *O. cornuta* 6 |
| LC792109/LC792170/- | -/R17 | Iran:Chaharmahal-e Bakhtiari, Lordegan, Mozaffarian 57549 (TARI) | *O. cornuta* 50 |
| LC792062/LC792131/LC792198 | H5/R5 | Iran: Khorasan, Jajarm, Memriani & Zangooei 42388 (FUMH) | *O. cornuta* 8 |
| LC792059/LC792129/LC792196 | H7/R7 | Iran: Khorasan, Esfrayen, Joharchi & Zangooei 39843 (FUMH) | *O. cornuta* 11 |
| LC792060/LC792130/LC792197 | H14/R6 | Iran: Khorasan, Bojnurd, Faghihinia & Zangooei 20121 (FUMH) | *O. cornuta* 9 |
| LC792061/-/LC792231 | H22/R4 | Iran: Khorasan, Bojnurd, Memriani & Arjmandi 37453 (FUMH) | *O. cornuta* 5 |
| LC792063/LC792132/LC792199 | H20/R6 | Iran: Khorasan, Esfarayen, Assadli Hoseinzadeh 2021-28 (TMUH) | *O. cornuta* 10 |
| LC792043/LC792115/LC792182 | H17/R25 | Iran: Semnan, Ahovan pass, Kazempour-Osaloo et al 2009-5 (TMUH) | *O. cornuta* 76 |
| LC792052/LC792120/LC792187 | H8/R25 | Iran: Semnan, Shahmirzad, Tayebi 2020-29 (TMUH) | *O. cornuta* 73 |
| LC792053/LC792121/LC792188 | H8/R25 | Iran: Semnan, Shahmirzad Tayebi 2020-31 (TMUH) | *O. cornuta* 74 |
| LC792041/-/- | -/R25 | Iran: Semnan, Damghan, Kazempour-Osaloo et al. 2009-6 (TMUH) | *O. cornuta* 75 |
| AB854504^*^/LC792125/LC792192 | H17/R24 | Iran: Semnan, Bashm, Kazempour-Osaloo 2009-1(TMUH) | *O. cornuta* 63 |
| LC792040/-/- | -/R24 | Iran:Semnan, Assadi & Ranjbar 82001 (TARI) | *O. cornuta* 69 |
| LC792074/LC792141/LC792208 | H18/R18 | Iran:Yazd, Mirhosseini& Soltani 2009-2 (TMUH) | *O. cornuta* 51 |
| LC792066/LC792135/LC792202 | H11/R11 | Iran: Kerman, Jiroft, Sarbijan, Bibak 2021-35 (TMUH) | *O. cornuta* 20 |
| LC792042/LC792174/LC792232 | H1/R1 | Turkey: Turgut 1113 (GAZI) | *O. cornuta* 3 |
| -/LC792172/- | -/- | Iran: Kerman, Assadi & Miller, 25353 (TARI) | *O. cornuta* 78^♦^ |
| -/-/LC792229 | -/- | Iran:Rudehen, Kazempour Osaloo 2021-36 (TMUH) | *O. cornuta* 79^♦^ |
| LC792111/LC792177/LC792234 | H3/R3 | Afghanistan: Rechinger 32331(W) | *O. cornuta* subsp*. leptacantha* Rech.f. 4 |
| LC792110/LC792175/- | -/R20 | Iran: Kohgilouyeh-Boirahmad, Dehdasht, Assadi & Abouhamzeh 46543 (TARI) | *O. elymaitica* Boiss. & Hausskn. 53 |
| LC137032^*^/LC792176/LC792233 | H21/R21 | Iran: Kohgilouyeh-Boirahmad, Ludab, Mozaffarian 71259 (TARI) | *O. elymaitic* 54 |
| AB854512^*^/LC137152^*^/LC792178 |  | Spain: Podlech 68902 (MSB) | *Onobrychis viciifolia* Scop. |
| AB911419^*^/LC792113/LC792180 |  | Iran: Kazempour Osaloo et al 2012-2a (TMUH) | *Onobrychis* *shahpurensis* Rech.f. |
| AB911418^*^/LC792112/LC792179 |  | Iran: Kazempour Osaloo et al. 2012-1 (TMUH) | *Onobrychis carduchorum* C.C.Towns. |
| AB329692^*^/-/- |  | Iran: Semnan, Freitag & Mozaffarian, 28397  (TARI) | *Eversmannia subspinosa* (Fisch. Ex DC.) B.Fedtsch*.* |
| AB854478^*^/-/- |  | China: Xu et al., 86862 (MSB) | *Corethrodendron scoparium* (Fisch. & C.A. Me) Fisch. & Basiner |
| AB854487^*^/-/- |  | Podlech 48626 (MSB) | *Greuteria argyrea* (Greuter & Burdet) Amirahm. & Kaz.Osaloo |
| LC137024^*^/-/- |  | Iran: Faghihnia and Zangooii 26074  (FUMH) | *Onobrychis arnacantha* Bunge ex Boiss |
| LC137044^*^/-/- |  | Afghanistan: Podlech 288872 (MSB) | *Onobrychis microptera* Baker ex Aitch. |
| LC388084^*^/-/- |  | Iran: Kazempour-Osaloo et al. 2012-12 (TMUH) | *Onobrychis* *gaubae* Bornm. |
| LC137039^*^/-/- |  | Iran: Rechinger 42530 (MSB) | *Onobrychis* *lunata* Boiss. |
| LC137020^*^/-/- |  | France: Auriault 16177 (MSB) | *Onobrychis* *aequidentata* (Sam.) d’Urv. |
| LC137027^*^/-/- |  | Iran: Kazempour Osaloo et al. 2012-4  (TMUH) | *Onobrychis* *caput*-*galli* Lam. |
| LC749438^*^/-/- |  | Iran: Assadi & Vosugi 24673 (TARI) | *Onobrychis* *cyri* Grossh. |
| LC749450^*^/-/- |  | Iran: Mozaffarian 46064 (TARI) | *Onobrychis* *luristanica* Rech.f. |
| LC749457^*^/-/- |  | Iran: Azarbaijan, Kazempour Osaloo  et al. 2008 (TMUH) | *Onobrychis* *persica* Širj. & Rech.f. |
| LC749445^*^/-/- |  | Turkey: Sorger 1992-0012580 (W) | *Onobrychis gracilis* Besser. |
| LC749449^*^/-/- |  | Russia: Vlasov s.n. 1985-0007286(W) | *Onobrychis* *iberica* Grossh. |
| AB911416^*^/-/- |  | Yugoslavia: [Macedonia](https://www.google.com/search?sca_esv=9928f787bd8332ef&rlz=1C1ASUM_enIR1058IR1058&sxsrf=ACQVn08bwqSEkGuYnxz0MQJT-jZkprWXUw:1708615609888&q=Macedonia&spell=1&sa=X&ved=2ahUKEwjgmvCTob-EAxWF8LsIHQP6AJQQkeECKAB6BAgJEAI), Podlech 28272 (MSB) | *Onobrychis* *alba* (Waldt. & Kit) Desv. |
| LC749429^*^/-/- |  | Turkey: Sorger 1992-0012458 (W) | *Onobrychis* *argaea* Boiss & Balansa |
| LC749464^*^/-/- |  | Turkey: Gunes s.n. (GAZI) | *Onobrychis* *stenostachya* Freyn |
| LC749448^*^/-/- |  | Spain: Laínz .s.n. 1958-0002286 (W) | *Onobrychis* *humilis* (Loefl.) G.López |
| LC137055^*^/-/- |  | France: Podlech 57824 (MSB) | *Onobrychis* *supina* DC. |
| LC749444^*^/-/- |  | Kyrgyzstan: Khokhryakov s.n. 1963-  0014392 (W) | *Onobrychis ferganica* (Širj.) Grossh. |
| LC749441^*^/-/- |  | Italy: Podlech 96960 (MSB) | *Onobrychis echinata* (Guss.) G.Don |
| LC749455^*^/-/- |  | Italy: Burri & Krendl s.n. 1994-0006105 (W) | *Onobrychis montana* DC. |
| LC137056^*^/-/- |  | China: Xu et al., 87212 (MSB) | *Onobrychis tanaitica* Spreng. |
| AB854508^*^/-/- |  | Iran: Ghahraman et al. 27318 (TUH) | *Onobrychis pulchella* Schrenk |
| LC137051^*^/-/- |  | Iran: Mozaffarian 93762 (TARI) | *Onobrychis sosnowskyi* Grossh. |
| LC137029^*^/-/- |  | Iran: Kazempour Osaloo et al. 2012-3 (TMUH) | *Onobrychis* *crista-galli* Lam. |

(^-^) not available in GenBank, (*) retrieved from GenBank. (^♦^) These accessions were not included in the analyses because of lacking nrDNA ITS sequence. The sample number for the accessions of *O. cornuta* was denoted beside its name.

Abbreviations used in plant accession information: FMUH, Ferdowsi University of Mashhad Herbarium, Mashhad, Iran, GAZI, Gazi University Herbarium, Ankara, Turkey, HUI, Herbarium of University of Isfahan, MSB Herbarium of Ludwig-Maximilians-Universitat, Munchen, Germany, SFAHAN, Herbarium of Isfahan Agricultural and Natural Resources Research and Education Center, Isfahan, TARI, Herbarium of Research Institute of Forests and Rangelands, Tehran, Iran, TMUH Tarbiat Modares University Herbarium, Tehran, Iran, TUH Tehran University Herbarium, Tehran, Iran, W, Museum of Natural History Vienna, Vienna, Austria. WANRCH West Azerbaijan Natural Resource Research Center Herbarium, Urmia, Iran.
