# Supplementary material for: Evolutionary history of an Irano-Turanian cushion-forming legume (Onobrychis cornuta)
Source: BMC Plant Biol. 2024 Mar 20;24:204. doi: 10.1186/s12870-024-04895-y (PMC10953250; doi:10.1186/s12870-024-04895-y)
Supplement: Supplementary file 2 — Supplementary Material 2 [file 12870_2024_4895_MOESM2_ESM.docx]

**Table S2** Dataset and tree statistics from separate and combined analyses of the nuclear and chloroplast regions.

| Combined  (nr+cp) | plastid (*rpl*32-*trn*L_(UAG)_+ *trn*T_(UGU)_-*trn*L_(UAA)_) | nrDNA ITS |  |
| --- | --- | --- | --- |
| 49 | 62 | 80 | Number of sequences |
| 2855 | 2188 | 659 | Nucleotide sites |
| 147 | 118 | 70 | Informative characters |
| 2708 | 2070 | 589 | Uninformative characters |
| 0.743 | 0.875 | 0.886 | CI of MPTs |
| 0.90 | 0.965 | 0.962 | RI of MPTs |
| K3Pu+F+I+G4 | TIM+F+G4 | TIM3+F+G4 | Evolutionary model selected (under AIC) |
